# Bone Density and Structure in Overweight Men With and Without Diabetes

**DOI:** 10.3389/fendo.2022.837084

**Published:** 2022-03-10

**Authors:** Jakob Starup-Linde, Marie Juul Ornstrup, Thomas Nordstrøm Kjær, Simon Lykkeboe, Aase Handberg, Søren Gregersen, Torben Harsløf, Steen Bønløkke Pedersen, Peter Vestergaard, Bente Lomholt Langdahl

**Affiliations:** ^1^ Department of Endocrinology and Internal Medicine, Aarhus University Hospital, Aarhus, Denmark; ^2^ Steno Diabetes Center Aarhus, Aarhus University Hospital, Aarhus, Denmark; ^3^ Steno Diabetes Center North Jutland, Aalborg University Hospital, Aalborg, Denmark; ^4^ Department of Clinical Biochemistry, Aalborg University Hospital, Aalborg, Denmark; ^5^ Department of Clinical Medicine, The Faculty of Medicine, Aalborg University, Aalborg, Denmark; ^6^ Department of Endocrinology, Aalborg University Hospital, Aalborg, Denmark

**Keywords:** diabetes, metabolic syndrome, bone turnover (markers), bone mineral density, HRpQCT

## Abstract

**Objective:**

Metabolic syndrome (MetS), type 1 diabetes (T1D), and type 2 diabetes, are associated with an increased risk of fractures; however, the impact of obesity on bone deficits in diabetes is unknown. We aimed to compare markers of bone structure, bone density, and bone turnover in non-diabetic overweight men with MetS and overweight men with T1D or T2D.

**Methods and Research Design:**

In this cross-sectional study we included participants from two previously described study cohorts consisting of participants with diabetes and participants with MetS. Participants underwent dual-energy X-ray absorptiometry measuring areal bone mineral density (aBMD) at the hip and lumbar spine, High Resolution peripheral Quantitative (HRpQCT) scan of the tibia and radius and measurement of circulating bone turnover markers. We compared groups with unpaired t test and performed multiple linear regression with adjustment for age, body mass index, and smoking.

**Results:**

We included 33 participants with T1D, 25 participants with T2D, and 34 participants with MetS. Bone turnover markers levels were comparable between T1D and MetS. aBMD at the hip was lower in T1D compared to MetS, also after adjustment. P1NP and Osteocalcin levels were lower among individuals with T2D compared to MetS, whereas aBMD were similar between the groups after multiple adjustments. We observed no difference in volumetric BMD at the tibia or radius between MetS and T1D and T2D, respectively. Participants with T2D had a higher trabecular number and lower trabecular separation compared to individuals with MetS at the tibia, which remained signficant after multiple adjustments.

**Conclusion:**

In conclusion, we observed no clinically important differences in bone density or structure between men with T2D, T1D, or MetS. However, men with T2D displayed lower bone turnover compared to MetS highlighting that T2D per se and not obesity, is associated with low bone turnover.

## Introduction

The incidence of bone fracture is expected to increase in the coming years as the prevalence of diabetes increases, and diabetes *per se* is associated with an increased risk of fracture, e.g., a meta-analysis reported a 7-fold and 1.4-fold increased risk of hip fracture for type 1 diabetes (T1D) and type 2 diabetes (T2D), respectively (1) and another meta-analysis reported a 1.2-fold increased risk for non-hip, non-vertebral fractures for T2D and a non-significant 1.9-fold increased risk for T1D (2).

The mechanisms of the increased fracture risk in patients with T1D and T2D are not fully elucidated. Metabolic syndrome (MetS), which consists of obesity, abdominal adiposity, increased blood pressure, impaired glucose tolerance, and dyslipidemia (3), is an important risk factor for T2D, and is associated with an increased risk of fracture in some studies, whereas other studies show neutral or even beneficial effects of obesity (4–6). Several factors may influence fracture risk in MetS and an observational study reported a strong association between MetS and increased fracture risk in individuals with increasing number of MetS characteristics (7). Impaired glucose tolerance is linked to insulin resistance which is associated with a lower bone turnover (8); however, in observational studies that excluded individuals with diabetes neither insulin resistance or impaired glucose tolerance are associated with an increased fracture risk (9, 10). Obesity is associated with a decreased risk of fracture at the hip and spine, but conversely associated with an increased risk for humerus-, femoral-, and ankle fractures (6, 11). Thus, the fracture patterns in T1D, T2D or overweight individuals are not similar. Bone mineral density (BMD) is increased in T2D and in overweight and obese individuals, whereas BMD is decreased in T1D (1, 12). However, in neither T1D, nor T2D, do the BMD levels explain the observed fracture risk. Low-grade inflammation is associated with insulin resistance (13), and insulin resistance and hyperglycemia may suppress bone turnover (14), which may in turn increase BMD but with a more brittle bone structure. Individuals with T1D and T2D have impaired bone formation which is likely to be a contributing factor to increased fracture risk (15).

Micro architectural changes in the cortical bone, as assessed by High Resolution peripheral Quantitative (HRpQCT), are suggested to explain the excess fracture risk in T2D; however, studies comparing microarchitecture in patients with T2D with a non-diabetic reference consist of patients with large differences in age and BMI (16–18). In a study from our group we found no difference in HRpQCT parameters between patients with T1D and T2D (19); however, another study reported lower vBMD in patients with T1D and microvascular complications compared to healthy controls with similar age, gender and BMI (20). The HRpQCT provides segmentation of the bone at the micro architectural level, permitting detection of trabecular or cortical deficits. In the present explorative study we aimed to compare markers of bone structure, bone density, and bone turnover in non-diabetic overweight men with metabolic syndrome (MetS) and overweight men with T1D or T2D. Due to the design of the study it is hypothesis generating.

## Materials and Methods

The study was performed according to the Helsinki Declaration and was approved at the Central Region Jutland Ethics Committee (1-10-72-5-13 and M-20110111).

In this cross-sectional study we included participants from two previously described study cohorts consisting of participants with diabetes and participants with MetS (21, 22). The participants with diabetes were included from outpatient clinics at the Aalborg and the Aarhus University Hospitals and participants with MetS were recruited by advertisements in local newspapers. We included men with diabetes and men with MetS between 50 and 65 years and with a body mass index (BMI) ≥ 25kg/m^2^. For participants with diabetes the HbA1c was above 49 mmol/mol within the last year, whereas participants with MetS were identified according to the International Diabetes Federation criteria (3): Central obesity (Waist circumference ≥94 cm and/or BMI ≥30 kg/m^2^) plus any two of the following: raised triglycerides (≥1.7 mmol/l), reduced high-density lipoprotein (≤1.03 mmol/l), raised blood pressure (≥systolic 130 mm Hg or diastolic 85 mmHg), and/or raised fasting plasma glucose (≥5.6 mmol/l). 61% (n = 21) of the participants with MetS displayed increased fasting glucose levels. We excluded individuals with renal impairment, bone metabolic and/or dysregulated thyroid disease and individuals treated with antiepileptics, glucocorticoids, lithium, or estrogen. Participants with MetS did not have a diagnosis of diabetes.

### Measurements

Participants underwent examinations at the Aalborg or the Aarhus University Hospitals. Areal BMD (aBMD) and bone mineral content (BMC) at the lumbar spine (L1–L4) and hip were measured by dual-energy X-ray absorptiometry using Hologic Discovery or Lunar prodigy scanners. Coefficient of variation (CV) and differences in measurement were evaluated by a repeatedly scanned Hologic Discovery phantom. The intrascanner precision CVs were 1% for both Hologic Discovery and Lunar Prodigy scanners. The Hologic Discovery scanner with most individuals scanned was selected as reference. The Lunar Prodigy scanners measured BMD significantly higher than the reference (14 and 15%, respectively), and the other Hologic Discovery scanner measured 2% higher, which was statistically significant. BMD was recalculated based on the conversion factors. T-scores were calculated based on extrapolation of the results reported by Kelly (23).

To assess bone geometry and -microarchitecture a subset of participants (i.e., participants recruited at the Aarhus University Hospital) were examined with HRpQCT (Xtreme CT; Scanco Medical) at the radius and tibia. A standard operating procedure was followed. Participants with diabetes were scanned on the right limb (unless there was a fracture) whereas participants with MetS were scanned on the non-dominant limb (unless there was a fracture). The tibia could not be scanned in two patients and the radius in four patients due to too large limbs or poor quality of the images. For each scan a two-dimensional scout view was performed to define the measurement region, using a threshold of 9.5 and 22.5 mm for the radius and tibia, respectively. At each site 110 images were obtained. All images were graded based on manufacturers suggestions from 1 to 5 (1 best, 5 worst), and images graded 4 or 5 required a rescan. Scans were analyzed with software provided by Scanco. Standard evaluation analysis and finite element analysis were performed. To ensure repeatability, all scans and evaluations were performed by JS-L or MO. The precision CVs were 0.7 and 1% for the tibia and radius, respectively.

Blood samples were collected in a non-fasting state for participants with diabetes and in a fasting state for participants with MetS, thus, we do not report estimates of C-terminal cross-linked telopeptide of type I collagen (CTX), which is influenced by fasting state (24). Procollagen type 1 amino terminal propeptide (P1NP) and osteocalcin are independent of food intake and were analyzed (25, 26). The manufacturer provided CVs for p-P1NP and p-osteocalcin (Roche Diagnostics, Mannheim, Germany) were <4 and <2% respectively. p-P1NP and p-osteocalcin were measured in two batches and at two sites, the Aalborg University Hospital and the Aarhus University Hospital. To ensure comparability, 10 samples were analyzed at both sites and showed high correlation and Bland–Altman plots revealed no systematic bias between sites. We obtained EDTA-stabilized blood for measurement of glycated hemoglobin (HbA1c) in individuals with diabetes. All measurements were performed in clinical biochemical laboratories accredited according to ISO 15189.

At the time of inclusion, the patients were interviewed by a medical doctor for medication and lifestyle history. Height and weight were measured, and a BMI was calculated. Diabetes duration was calculated from the year of diagnosis of diabetes to the year of the examination. Smoking was grouped as current smokers, previous smokers, and non-smokers. Microvascular complications were grouped as of nephropathy, retinopathy, and neuropathy.

### Statistics

Normality of data was checked by Q–Q plots, and equal variance between groups was assessed by Bartlett’s test. Data are presented as means and 95% confidence interval. We compared overweight individuals with MetS, T1D and T2D with an oneway ANOVA and if the global null hypothesis is rejected the applied Fishers protected least significant difference test was applied to detect differences between the specific groups.

We adjusted for age, BMI, and smoking in a multiple adjusted linear regression. The following assumptions were checked: assumption of normal distribution, assumption of linearity between dependent and independent variable, assumption of reliability, and assumption of homoscedasticity

No power calculation was performed as the present study is a *post hoc* analysis of previous studies, thus the study is an explorative study.

STATA 17 (Stata Corp) was used to perform the statistics.

## Results

We included 33 participants with T1D, 25 participants with T2D, and 34 participants with MetS. Characteristics of the subjects are displayed in **Table 1**.

**Table 1:** Characteristics, dual-energy X-ray parameters and bone turnover markers of included subjects.

	Type 1 diabetes (n = 33)	Type 2 diabetes (n = 25)	Metabolic syndrome (n = 34)
Age (years)	57.9 (55.5; 58.5)*	58.1 (56.5; 59.7)*	54.9 (53.9; 55.9)
BMI (kg/m^2^)	28.3 (27.4; 29.1)*^£^	32.0 (30.9; 33.1)	33.1 (31.7; 34.4)
Plasma creatinine (umol/l)	82.4 (76.6; 88.3)	78.4 (71.1; 85.8)	79.1 (76.3; 81.9)
Plasma glucose (mmol/l)	10.0 (9.2; 11.8)*^£^	11.2 (10.6; 13.6)*	5.82 (5.61; 6.03)
Hba1c (mmol/mol)	64.1 (60.5; 67.7)	65.8 (61.3; 70.4)	
Diabetes duration (years)	25.3 (21.3; 29.3)^£^	12.4 (10.1; 14.7)	
Microvascular complication (%)	45.4 (27.5; 63.3)	48.0 (27.0; 69.0)	
Neuropathy (%)	21.2 (6.5; 35.9)	48.0 (27.0; 69.0)	
Retinopathy (%)	45.5 (27.5; 63.4)	16 (0.5; 31.4)	
Smoking			
Current (n)	15 (45% of total)	11 (44% of total)	18 (53% of total)
Previous (n)	14 (42% of total)	8 (32% of total)	13 (38% of total)
*Never (n)*	4 (12% of total)	6 (24% of total)	2 (6% of total)
**Bone turnover markers**			
P1NP (ng/ml)	37.8 (34.3; 41.4)	31.2 (27.1; 35.4)*	41.8 (37.5; 46.2)
Osteocalcin (ng/ml)	17.1 (15.6; 18.6)*^£^	13.0 (11.0; 14.9)*	19.3 (17.8; 20.9)
**DXA**			
aBMD lumbar spine (g/cm^2^)	1.07 (1.03; 1.12)	1.15 (1.07; 1.23)	1.05 (1.00; 1.10)
aBMD hip (g/cm^2^)	0.97 (0.92; 1.01)*	1.03 (0.99; 1.08)	1.04 (1.00; 1.09)
aBMD femoral neck (g/cm^2^)	0.82 (0.78; 0.86)	0.84 (0.80; 0.88)	0.82 (0.79; 0.86)
aBMD forearm (g/cm^2^)	0.64 (0.61; 0.66)	0.63 (0.61; 0.66)	0.63 (0.61; 0.64)

*Statistical significantly different when comparing with the MetS group using oneway ANOVA and Fishers protected least significant difference (p < 0.05).

^£^Statistical significantly different when comparing with the T2D group.

Data are presented as means and 95% confidence intervals unless otherwise specified.

Participants with T1D or T2D were slightly older than the individuals with MetS (57.9 years, 95% CI 55.5; 58.5 and 58.1 years, 95% CI: 56.5; 59.7 vs. 54.9 years, 95% CI 53.9; 55.9). Participants with T1D had a lower BMI compared to individuals with MetS (28.3 kg/m^2^, 95% CI: 27.4; 29.1 vs. 33.1 kg/m^2,^95% CI: 31.7; 34.4), whereas participants with T2D and MetS were comparable. As expected, the plasma glucose levels were higher among individuals with T1D or T2D compared to MetS (10.0 mmol/l,95% CI: 9.2; 11.8 and 11.2 mmol/l, 95% CI: 10.6; 13.6 vs. 5.82 mmol/l,95% CI: 5.61; 6.03). The mean HbA1c at time of investigation was 64.1 and 65.8 mmol/mol for individuals with T1D and T2D, respectively. Almost 50% of the individuals with diabetes had at least one microvascular complication (45.5 and 48.0% for T1D and T2D, respectively). We observed no significant difference in p-creatinine levels or smoking duration between groups. Among the individuals with T2D all 25 subjects used metformin. Four used sulfonylureas, fourteen used glucagon like peptide-1 (GLP-1) receptor agonists, two used dipeptidylpeptidase-IV (DPP-IV) inhibitors, three used sodium glucose transporter 2 (SGLT-2) inhibitors, and one used insulin. Within the group of subjects with T2D we observed no difference in bone turnover markers or bone density between those using and not using GLP-1 receptor agonists.

### Bone Turnover Markers

P1NP levels were comparable between T1D and MetS. Osteocalcin was significantly lower in T1D (17.1 ng/ml, 95% CI: 15.6;18.6 vs. 19.3 ng/ml, 95% CI: 17.8;20.9) however, the difference did not remain after adjustment for age, BMI, and smoking. P1NP (31.2 ng/ml, 95% CI: 27.1; 35.4 vs. 41.8 ng/ml, 95% CI: 37.5; 46.2) and osteocalcin (13.0 ng/ml, 95% CI: 11.0; 14.9 vs. 19.3 ng/ml, 95% CI: 17.8; 20.9) levels were lower among individuals with T2D compared to MetS, also after adjustment for age, BMI, and smoking. Fasting glucose levels in individuals with MetS were not associated with P1NP or osteocalcin levels.

### Bone Density

aBMD at the hip was lower in T1D compared to MetS (0.97 g/m^2^, 95% CI: 0.92,1.02 vs. 1.04 g/m^2^, 95% CI: 1.00; 1.09), also after adjustment for age, BMI, and smoking, but with no difference at the lumbar spine. aBMD at the lumbar spine was borderline higher in T2D compared to MetS (p = 0.05 in the global ANOVA test), (1.15 g/m^2^, 95% CI: 1.07;1.23 vs. 1.05 g/m^2^, 95% CI: 1.00; 1.19), but with no difference at the hip, however the difference was abolished after adjustment for age, BMI, and smoking. Results from the HRpQCT scans are displayed in **Tables 2**, **3**.

**Table 2:** Results from high resolution peripheral quantitative CT of the tibia.

	Type 1 diabetes (n = 16)	Type 2 diabetes (n = 17)	Metabolic syndrome (n = 32)
Structural parameters			
Cortical area (mm)	151 (135; 166)	164 (150; 177)	164 (155; 172)
Trabecular area (mm)	741 (686; 796)	774 (691; 857)	769 (714; 825)
Cortical perimeter (mm)	121 (116; 125)	123 (117; 128)	122 (118; 125)
Cortical thickness (mm)	1.25 (1.14; 1.36)	1.35 (1.21; 1.48)	1.36 (1.27; 1.46)
Bone volume ratio (%)	16.0 (14.7; 17.3)	16.6 (15.2; 18.0)	15.5 (14.7; 16.3)
Trabecular number (per mm)	2.13 (2.00; 2.28)	2.32 (2.18; 2.46)*	2.06 (1.96; 2.15)
Trabecular thickness (mm)	0.08 (0.07; 0.08)	0.07 (0.07; 0.08)	0.08 (0.07; 0.08)
Trabecular separation (mm)	0.40 (0.37; 0.43)	0.37 (0.34; 0.39)*	0.42 (0.39; 0.44)
Density parameters			
vBMD (mg HA/cm^3^)	309 (291; 327)	321 (299; 344)	313 (298; 330)
Cortical vBMD (mg HA/cm^3^)	856 (838; 873)	857 (836; 878)	870 (856; 883)
Trabecular vBMD (mg HA/cm^3^)	192 (176; 208)	199 (182; 216)	186 (176; 196)
Metatrabecular vBMD (mg HA/cm^3^)	252 (236; 268)	261 (245; 279)	249 (238; 259)
Inner trabecular vBMD (mg HA/cm^3^)	152 (135; 169)	156 (139; 173)	143 (134; 153)
Finite Element Analysis			
Tissue Stiffness (kN/mm)	268 (223; 313)	314 (278; 350)	294 (284; 304)

*Statistical significantly different when comparing with the MetS group using oneway ANOVA and Fishers protected least significant difference (p <0.05).

Data are presented as means and 95% confidence intervals.

**Table 3:** Results from high resolution peripheral quantitative CT of the radius.

	Type 1 diabetes (n = 15)	Type 2 diabetes (n = 18)	Metabolic syndrome (n = 34)
Structural parameters			
Cortical area (mm)	73.0 (65.4; 80.7)	75.9 (67.8; 83.9)	77.5 (71.9; 83.0)
Trabecular area (mm)	311 (278; 344)	334 (304; 364)	330 (309; 351)
Cortical perimeter (mm)	86.5 (82.5; 90.6)	88.9 (85.6; 92.2)	88.8 (86.4; 91.1)
Cortical thickness (mm)	0.85 (0.75; 0.95)	0.86 (0.76; 0.96)	0.88 (0.81; 0.94)
Bone volume ratio (%)	14.9 (13.0; 16.8)	14.3 (13.0; 15.6)	15.1 (14.1; 16.0)
Trabecular number (per mm)	2.18 (1.96; 2.39)	2.24 (2.11; 2.37)	2.17 (2.09; 2.26)
Trabecular thickness (mm)	0.07 (0.06; 0.08)	0.06 (0.06; 0.07)	0.07 (0.07; 0.07)
Trabecular separation (mm)	0.41 (0.35; 0.47)	0.39 (0.36; 0.42)	0.40 (0.38; 0.42)
Density parameters			
vBMD (mg HA/cm^3^)	322 (292; 353)	314 (290; 338)	324 (306; 342)
Cortical vBMD (mg HA/cm^3^)	864 (840; 888)	861 (832; 890)	869 (853; 884)
Trabecular vBMD (mg HA/cm^3^)	179 (156; 202)	172 (156; 188)	181 (170; 193)
Metatrabecular vBMD (mg HA/cm^3^)	239 (218; 260)	232 (216; 247)	240 (229; 253)
Inner trabecular vBMD (mg HA/cm^3^)	137 (113; 162)	131 (114; 148)	140 (129; 151)
Finite Element Analysis			
Tissue Stiffness (kN/mm)	113 (103; 124)	116 (109; 124)	119 (112: 126)

*Statistical significantly different when comparing with the MetS group using oneway ANOVA and Fishers protected least significant difference (p <0.05). Data are presented as means and 95% confidence intervals.

We observed no difference in vBMD at the tibia or radius between MetS and T1D and T2D, respectively.

### Bone Microarchitecture

We found no difference in tissue stiffness, a marker of bone tissue strength or cortical parameters. There were no differences in trabecular parameters or cortical parameters between MetS and T1D at the radius or tibia.

Regarding trabecular parameters, participants with T2D had a higher trabecular number (2.32 per mm, 95% CI: 2.18; 2.46 vs. 2.06 per mm, 95% CI: 1.96; 2.15) and lower trabecular separation (0.37 mm, 95% CI: 0.34; 0.39 vs. 0.42 mm, 95% CI: 0.39; 0.44) compared to individuals with MetS at the tibia, which remained significant after adjustment for age, BMI, and smoking. These findings were not present at the radius. Neither cortical thickness, trabecular thickness, cortical perimeter nor cortical vBMD differed between the T2D and MetS at the radius or tibia.

## Discussion

In the present study we found that the circulating bone formation markers, P1NP and osteocalcin, were lower in T2D compared to weight matched individuals with MetS, whereas we found no difference in aBMD between T2D and MetS. T2D, T1D and MetS are all characterized as low bone turnover conditions where especially bone formation markers are decreased (15, 27–29). We have previously shown that bone turnover markers are lower in T2D compared to T1D (21), and our present findings highlights that individuals with T2D also have a lower level of circulating bone formation markers compared to MetS. A recent study similarly showed that overweight women with T2D displayed lower bone turnover compared to BMI matched healthy controls (30).

Individuals with T1D and T2D had relatively long diabetes duration, were rather glycemic dysregulated, and 50% had a microvascular complication. This demonstrates that the diabetes patients are affected by their disease and this may explain the differences we have observed. We observed indications of altered trabecular structure at the tibia in T2D compared to MetS, with an increased trabecular number and a lower trabecular spacing. The trabecular number is the number of trabeculae per unit of length and a lower spacing indicate shorter distances between the trabeculae which may indicate denser bone. Thus, the observed increased trabecular number and a lower trabecular spacing may reflect that T2D is a condition with a relatively high BMD and low bone turnover, however it may also be a chance finding. Besides the differences in trabecular structure, we observed no differences in vBMD, tissue stiffness, or measures of bone microarchitecture between groups. Samelson and colleagues investigated 129 patients with T2D and 940 control subjects (17). In accordance with our results they reported an increased trabecular number (p = 0.09) and a reduced trabecular spacing (p = 0.16) at the tibia in patients with T2D. In their study, 14% of the included individuals with T2D used glitazones which decreases bone mass and 10% of the controls used estrogens which *increases* bone mass. These two factors level the results between patients and controls and explain why their results were only borderline significant. Shanbhogue and colleagues, however, reported similar trabecular spacing and number between individuals with or without T2D, however T2D individuals were significantly more obese (18).

It is hypothesized that the increased fracture risk in T2D may be caused by an accumulation of micro fractures due to low bone turnover (31). This may explain the paradox of seemingly high aBMD and yet a high fracture rate. Additionally, this may provide insights on the observed differences in fracture rates and fracture sites between T2D and obesity or MetS, which are not explained by aBMD and vBMD measurement or bone microarchitecture.

Several drugs are used in the treatment for T2D, but only glitazone (which was not used by any of the participants in the present study) shows detrimental effects, whereas it is controversial whether other antidiabetic drugs influence fracture risk, bone density, or bone turnover (32). Metformin has in a randomized controlled trial been shown to reduce bone turnover markers (33), whereas observational studies in general report either beneficial or neutral effects of metformin on fractures or BMD (32, 34). Sulfonylurea and insulin do not change bone turnover (32, 35). The effect of these drugs on fracture risk is controversial as both drugs have been linked to an increased fracture risk through an increased risk of hypoglycemia, however, observational studies report neutral or even protective effects of the treatments (34). DPP-IV and GLP-1 receptor agonist have in preclinical studies shown bone formative effects (32), but observational and randomized controlled trials showed neutral effects on fracture risk (34). In spite of these findings, GLP-1 receptor agonists seem to reduce bone resorption and hip BMD decrease during weight loss in T2D, which may be protective as weight loss is associated with an increased risk of fracture (36, 37). SGLT-2 inhibitors were initially suspected to increase fracture risk due to an increased urinary excretion of calcium, however, recent observational and randomized controlled trials showed neutral effects (32). Our findings suggests that T2D is a state of low bone turnover which is not explained by overweight or other parts of MetS, but may due to either glucose fluctuations (38) or more likely could be caused by insulin resistance, as bone turnover markers are lower compared to individuals with T1D. In abdominal adipose individuals without diabetes, P1NP was inversely associated with insulin resistance (8, 29). In obese insulin resistant subjects, bone turnover markers were lower compared to obese insulin-sensitive subjects (14). Furthermore, it is hypothesized that the osteoblast may become insulin resistant, which may explain a reduction in bone formation (39). In T2D, sclerostin, a product of the osteocytes which impairs bone formation and stimulate bone resorption (15), is increased, however, it is unknown whether osteocytes are influenced by insulin resistance.

The strength of the present study is that the MetS group is relatively similar to the T1D and T2D groups regarding age and BMI although small differences were present. Study participants were consecutively recruited.

This makes our findings less susceptible to the influence on bone by factors as, e.g., age-related loss, arthrosis, and increased mechanical loading by adiposity. On the other hand, our selection reduced the number of eligible individuals within the study, and thus we may be underpowered to detect differences. However, we observed no trends toward altered microarchitecture or density in T1D or T2D compared to MetS. For the HRpQCT measurement we scanned the right limb for individuals with diabetes and the non-dominant limb for individuals with MetS, which may provide better micro-architectural parameters for the diabetes groups. We did not observe any trend toward poor microarchitecture in individuals with diabetes compared to MetS, and we do not believe this influenced our results.

We excluded CTX analyses from this study as individuals with diabetes were not fasting at the time of blood sample. P1NP and osteocalcin are markers that are stable and only small reduction of up to 10% are observed following a meal (24–26, 40), which would not influence our results.

In conclusion, we observed no clinically important differences in bone density or structure between men with T2D, T1D, or MetS. However, men with T2D displayed lower bone turnover compared to MetS highlighting that T2D per se and not obesity, is associated with low bone turnover. Future research should aim at elucidating the mechanisms for low bone turnover in T2D.

## Data Availability Statement

The datasets presented in this article are not readily available because the regulations of the Danish Data Protection Agency applies. Requests to access the datasets should be directed to the corresponding author.

## Ethics Statement

The studies involving human participants were reviewed and approved by the Central Region Jutland Ethics Committee, Denmark. (1-10-72-5-13 and M-20110111). The patients/participants provided their written informed consent to participate in this study.

## Author Contributions

JS-L, MØ, TNK, SL, AH, SG, TH, SBP, PV, and BLL conceptualized and designed the study. JS-L, MØ, and TNK recruited the participants. JS-L and MØ conducted the analyses and drafted the manuscript. JS-L, MØ, TNK, SL, AH, SG, TH, SBP, PV, and BLL interpreted the data, and revised the manuscript critically. JS-L, MØ, TNK, SL, AH, SG, TH, SBP, PV, and BLL approved the final version of the manuscript. All authors listed have made a substantial, direct, and intellectual contribution to the work and approved it for publication.

## Funding

This work was supported by the Danish Council for StrategicResearch (Grant number 10-0934999), the European Union Framework Programme 7 (Grant 282526), the Steno Collaborative grant, Novo Nordisk Foundation, Denmark (Grant no. NNF18OC0052064), and The AP Møller Foundation for the Advancement of Medical Science.

## Conflict of Interest

The authors declare that the research was conducted in the absence of any commercial or financial relationships that could be construed as a potential conflict of interest.

## Publisher’s Note

All claims expressed in this article are solely those of the authors and do not necessarily represent those of their affiliated organizations, or those of the publisher, the editors and the reviewers. Any product that may be evaluated in this article, or claim that may be made by its manufacturer, is not guaranteed or endorsed by the publisher.
